# Alternative Friendships to Improve Men’s Health Status. The Impact of the New Alternative Masculinities’ Approach

**DOI:** 10.3390/ijerph18042188

**Published:** 2021-02-23

**Authors:** Oriol Ríos-González, Mimar Ramis-Salas, Juan Carlos Peña-Axt, Sandra Racionero-Plaza

**Affiliations:** 1 Department of Pedagogy, Rovira i Virgili University, 43007 Tarragona, Spain; 2 Department of Sociology, University of Barcelona, 08034 Barcelona, Spain; mimarramis@ub.edu (M.R.-S.); sandraracionero@ub.edu (S.R.-P.); 3 Facultad de Ciencias Sociales y Humanidades, Instituto de Estudios Sociales y Humanísticos IDESH, Universidad Autónoma de Chile, 1090 Temuco, Chile; juan.pena@uautonoma.cl

**Keywords:** friendship, new alternative masculinities, solidarity, self-confidence, happiness

## Abstract

Background: Men who develop behaviors connected with the model of hegemonic masculinity present several health problems. Previous research has shown the types of problems that men commonly suffer in this regard such as chronic diseases, dietary disorders, and traffic accidents. To combat and overcome this situation, several campaigns, policies and recommendations have been undertaken, and consequently, their influence has been analyzed. However, there have been few investigations into the role of men’s friendship in the reduction of these physical health problems. The findings presented in this article are focused on this issue, illustrating the impact of male friendship on the shaping of healthy behaviors. Methods: Drawing upon a qualitative-based methodology articulated in a case study of the Men in Dialogue association, located in Spain, the study has followed the premises of the communicative approach, a total of 15 structured online open-ended questionnaires have been performed and analyzed. The median age of the participants is 37.5 years. Results: The findings show how men involved in Men in Dialogue are promoting a kind of masculine friendship that is improving men’s emotional well-being and, consequently, their physical health.

## 1. Introduction

Friendships play a critical role in people’s lives. They can influence the activities people engage in, how they relate to others, and their worldview [1]. However, for men, traditional gender norms may limit the benefits of close male relationships [2]. Thus, previous research suggests that toxic male friendship influences men to follow unhealthy habits, such as drug and alcohol abuse [3]. This article will analyze this problem; however, it will go further and show different strategies to prevent such problems through the promotion of transformative friendship [4,5,6] within the development of a case study focused on an egalitarian men’s group called Men in Dialogue, which was established in Spain.

Men in Dialogue is a men’s group that draws on the egalitarian men’s movement [7]. It was created in 2007, and its theoretical foundations are the ideals of new alternative masculinities—NAM [8]. Based on a review of findings obtained on eleven competitive research projects focused on gender-based violence, Flecha, Puigvert, and Rios defined NAM as “men who combine attraction and equality and generate sexual desire” [8] (p. 102) and who work for the eradication of gender-based violence. Men who follow NAM’s approach define themselves as egalitarian men with a strong positioning against gender inequalities. However, researchers highlight that NAM includes attractiveness as an issue to be considered for combating gender-based violence. Therefore, they affirm the important value of attractiveness because it contributes to overcoming the traditional dichotomous scheme that considers, on the one hand, “good boys” to be those who are ethical and responsible but normally do not awaken desire and, on the other hand, “bad boys” to be those who are engaged in violent behaviors but do awaken desire.

Contrarily, Flecha, Puigvert, and Rios [8] described the existence of two more ideal types that are antagonistic to NAM’s ideal type: dominant traditional masculinity (DTM) and oppressed traditional masculinity (OTM). DTM refers to men who are aggressive and violent against women and who, due to a chauvinist socialization process, are sometimes considered charismatic because of the existing link between violence and attractiveness. However, OTM are “good boys” who are not perceived as attractive but who also contribute to the aforementioned chauvinist socialization process by reproducing double-standard phenomena that connect goodness with boredom. This distinction framed on traditional models of masculinity is in line with previous analyses such as the one carried out by Levant [9]. Levant et al. [9] confirmed the presence of traditional masculinity ideology but went further and formulated a theoretical approaching of three varieties of male gender role strain. Thus, they differentiated between discrepancy-strain, dysfunction-strain, and trauma-strain. The first concerns men’s failure to live up to an internalized ideal of manhood. Dysfunctional type is characterized by responding to the requirements of traditional male codes, although these behaviors have negative effects on the men themselves. Finally, trauma strain refers to the male role socialization process, which men perceive as intrinsically traumatic.

International bodies such as the European Commission (EC) have expressed concerns about the abovementioned men’s health habits. For instance, in drawing on the conclusions of the report European men’s health [10], it is seen that men present a higher percentage of premature death rates than women. In this regard, the EC stresses the role played by lifestyle in the decline of health, which is strongly connected with practices such as “smoking, excessive alcohol consumption, physical inactivity and poor diet in the etiology of many of the principal causes of mortality and morbidity, including cardiovascular and respiratory diseases” [10,11]. More recently, the EC [12] published another report in which information on the situation of European men’s health is updated [12]. This report confirms the previous analyses and shows that men’s premature mortality is increasing and that this is strongly connected with several gendered practices; for example, the authors affirmed “the costs of masculinity”. Therefore, there is an important number of studies from Hispanic-American scholars in the framework of men’s studies that confirms the negative health consequences of reproducing these gendered practices linked to hegemonic masculinities [13].

Research on masculinity has widely investigated the social and psychological factors that drive men into this situation [13,14]. In this regard, there is evidence that psychological factors, including specific stressors and social issues such as gendered stereotyping, influence men’s health status, particularly in the promotion of various risk-taking behaviors [15,16]. Another research line in this field pays attention to men’s use of health services [17,18,19]. The findings in this line show that some men scarcely discover the use of the healthcare system relevant to them, which is on many occasions influenced by the gendered socialization that connects hegemonic masculinity with rude and selfish attitudes [20]. Along the same vein, the research has also paid attention to the role played by friendship in men’s health [2,21,22]. Most of these studies corroborate that negative masculine peer camaraderie reinforces the performance of several unhealthy practices [3].

Conversely, Flecha [4] shed light on this issue and has noted, on the one hand, the positive impact of real friendship on all aspects of life and, on the other hand, the potential transformation in people’s lives due to the movements connected to NAM [23,24]. With our article, we deepen the understanding of these benefits, specifically in regards to health, paying attention to how, through the rejection of hegemonic masculinity and the promotion of friendship and alternative models of masculinity, undesirable health behaviors can be combatted. In fact, analyses on hegemonic masculinity [25,26] have demonstrated the necessity of finding such alternative models because they conclude that masculinity includes the forming of a set of toxic relationships that drive men to follow risky practices.

Drawing on this basis, the article will further investigate this issue; thus, the article is divided into four sections: (1) the state of masculinity studies with a focus on the development of men’s friendships and men’s health status; (2) the applied methods, for which the procedure and data analysis are thoroughly explained; (3) the results of the current study, for which the exclusionary and transformative effects of friendship on men’s health are considered; and (4) the discussion and the scope of future research in the field.

### State of Masculinity Studies

The field of masculinity studies includes extensive analyses of how men are addressing their health issues and friendship [27,28]. Among different perspectives in relation to masculinity and health, there is a predominant topic that concerns the analysis of the factors that drive men to have high levels of health problems. First, we briefly introduce this aspect and men’s friendships and their connections with health-related risks are described.

The question of how men develop risk-taking behaviors that drive them to suffer health problems throughout their lives has been widely investigated [29,30]. In fact, this is a question that has been analyzed from different perspectives, including biological and psychological studies. In this regard, it is important to note the work developed by Harrison [31] in his paper, Warning: the male sex role may be dangerous to your health, in which he describes in detail how men’s life expectancy has been analyzed. He distinguishes between biogenetic and psychosocial perspectives. The first perspective bases its explanations about men’s mortality on genetic factors [32] and concludes that men are less biologically equipped than women. The latter perspective was developed by Jourard [33] and focuses on sex-role socialization as an explanatory factor in understanding men’s higher mortality rates.

Jourard’s perspective goes deeper into the influence of social dimensions in men’s psychological development. In fact, from this perspective, sex-role socialization is crucial to understanding risk-taking behaviors, such as drinking, dangerous driving, and unhealthy eating [16]. About this last aspect, there is research that underscores how stressors, such as chronic strain and traumatic events, influence men’s self-rated health [14]. In the same line, there are other analyses that pay attention to specific behaviors linked to a particular type of masculinity that work as barriers for men to overcome illnesses such as cancer. Thus, men feel embarrassed or fearful when faced with severe health problems and these types of attitudes are strongly linked with the maintenance of specific gender roles [34].

As introduced above, the investigation of gender roles has examined the reasons for the perpetuation of gender differences in areas such as health [17]. Raewyn Connell’s [25,35] conceptualization of hegemonic masculinity also considers this aspect. She established two differentiated gender categories: “hegemonic masculinity” and “emphasized femininity”. Based on a qualitative study performed in secondary schools from the perspective of Gramsci’s theorization of hegemony, Connell and colleagues [36] affirmed the existence of dominant gender models and, consequently, the fact that gender models subordinate others that are dissident. In relation to masculinity, the authors concluded that the hegemonic model reproduces negative behaviors linked to the exercise of power and violence.

Several years later, Connell and Messerschmidt [25] reformulated this concept and argued that social relations between men, as well as between men and women, have changed from the former definition. Emerging “protest masculinities”, in observing vulnerable groups (such as ethnic minorities) and the role that women play in current western societies, are questioning the existence of a unique model of hegemonic masculinity. In fact, there is significant evidence, starting with the current impact of globalization. This analysis drives her to differentiate about the role that sociocultural factors have in the shaping of different types of masculinities [36]. Similarly, Connell [37] has recently widened the conceptualization of hegemonic masculinity between two typologies of hegemonic masculinities, those that exercise violence and those that do not. Edwards [15] draws on this analysis to explore how health is influenced by constructs of masculinity and femininity. She concludes that gender and sexuality mediate people’s health status in western countries, reproducing the hegemony of a heterosexual and patriarchal system that defines health needs.

In a similar vein, there has been research that is focused on how men perceive illnesses, such as HIV and stress, as well as on how they view access to health services [19,38]. In their examination of masculinity and health beliefs, Mahalik and Burns [38] highlighted the importance of gender roles and social norms in shaping men’s health beliefs and their practices with respect to these beliefs (e.g., diet, sports, alcohol and tobacco use, medical checkups). They affirm that men increase their involvement in healthy practices when they have a normative connotation among other men. However, regarding how men make use of healthcare systems, the literature distinguishes between two types of discourse: biomedical and moral [20,39].

The first approach is used to construct medicine and healthcare as objective and rational elements that never consider the emotional dimension. According to this discourse, men’s health is defined in a framework of power relations where doctors, from a hierarchical position, establish the ways in which men should “understand, regulate and experience their bodies” [39] (p. 249). However, in the moral discourse [20], attention is paid to men’s public positioning toward health care. Thus, it is common to observe that men argue publicly that the use of the healthcare system is of primary importance to their well-being, yet there is important evidence that dominant forms of masculinity lead to infrequent use of such healthcare systems.

As mentioned, there is little research that goes deeper into the role played by friendship in men’s lives, particularly in the improvement of physical health. However, some analyses illustrate the shaping of practices that reinforce interactions in men’s friendships based on hegemonic masculinity. One of these practices refers to the lack of verbal expression of closeness within male friendships, which shows men’s difficulty deepening same-gender intimate relationships [40]. Along the same lines, but linked to the construction of toxic friendships, some studies identify the persistence of masculine normativity, which directly and indirectly contributes to men’s alcohol consumption. These studies suggest how peer influence is a predictor of this consumption because men, particularly young men, are socialized to follow hegemonic masculine behaviors such as playboy performance or emotional control [3]. Thus, men’s camaraderie, based on hegemonic masculinity, maintains a type of toxic relationship that drives them to perform practices that affect their health status.

From an analogous line of research, but introducing elements such as practicing physical exercise and being more health-oriented, other investigations make gender comparisons that show how women feel comfortable with behaviors that provide intimacy and peer support, but men share other moments with their same-sex friends that deliver positive feedback, such as the practice of physical exercise [22]. From this analysis emerges the idea that physical exercise with friends is perceived by men as an element that improves their emotional well-being. Connected with this finding is an important amount of research that focuses on the impact of social and friendship networks on the improvement of men’s health. In this regard, the evaluations developed around the effects of support networks in gay men with HIV/AIDS are particularly relevant; the findings illustrate that these networks become useful in reducing the advancement of these men’s illnesses [22,41]. In fact, it has been widely demonstrated that the social support given by peers can lead to a reduction in levels of stress and anxiety [42,43].

Social support is especially relevant in relation to gay men, who face discrimination for their sexual orientation, particularly if they have HIV/AIDS. From another perspective, but still with a focus on friendship and health issues, Emslie, Hunt, and Lyons [43] performed a qualitative study on straight men who drink alcohol. The results show that drinking beer is perceived as an act of friendship by men, but contrarily there is a different type of masculine friendship that uses soft drink consumption to foster emotional support.

The findings presented in this article are in line with findings which shed light on the existence of a type of alternative behavior that fosters better health habits in men. However, they go beyond providing new data on the effects of friendship, framed on an egalitarian men’s association, on men’s behavior, and in the improvement of physical health.

## 2. Materials and Methods

The methodology employed in the present study follows a qualitative approach focused on the analysis of how friendship and health are approached in an egalitarian men’s association. We have chosen this type of approach aimed at deepening the reflections and analyses constructed by men around the impact of friendship, which is framed in the Men in Dialogue men’s group, on health habits. In this regard, we have conducted a case study seeking to explore how interactions between men are shaping a type of alternative masculine friendship. Previous research has already quantified men’s beliefs and practices about health issues [30,44], but in the present investigation will provide knowledge about the connections of friendship and health habits.

To assure the validity of our study, two strategies that draw on the communicative approach were considered [45]. One strategy implies that researchers are responsible for informing the participants about the existing scientific knowledge on the topic being researched. We followed this procedure by presenting to the Men in Dialogue men’s group the previous research findings on this topic. Thus, particular attention was paid to Connell’s work [25,35,37], who is an author that participants in the study had previously discussed. In fact, Men in Dialogue’s meetings are focused on debates around scientific contributions connected with masculinity. For instance, members of the men’s group have read Tristan Bridges and CJ Pascoe’s [46] analysis of “hybrid masculinities”, Raewyn Connell’s [25] work on the theoretical development of “hegemonic masculinity” and Jesús Gómez’s [47] study on radical love and gender socialization. Our research team could perceive that these readings help participants to more properly understand the knowledge that we presented.

The second strategy concerns the commitment to empirical truth, which suggests that there is a shared interest between researchers and social actors in the study of solutions to social problems. This orientation is crucial in communicative methodology because its objective is to obtain knowledge that could, broadly speaking, transform society, which means changing the situation of exclusion experienced by the most disadvantaged groups, as well as converting inequalities or difficult situations that society as a whole—both privileged and disadvantaged groups—could face at any particular moment. Thus, communicative methodology is useful for both vulnerable and non-vulnerable populations; however, it is most beneficial to the first group, because they are commonly the most silenced group in a research context. Another communicative methodology premise refers to the lack of interpretative hierarchy between researchers and participants in the research. This is manifested when the main findings obtained through data analysis are contrasted with the participants involved in the investigation. More details on this matter will be described later in this section.

To conclude, all these specific procedures make a difference, methodologically speaking, and this approach has thus been adopted as very relevant at the international level. In fact, the procedures have been recognized by the European Commission as a useful methodological strategy to analyze social inequalities for achieving social impact [48,49].

### 2.1. Participants and Instruments

The case study developed an online open-ended questionnaire that was completed by 15 men involved or connected with Men in Dialogue. Men in Dialogue is based in Barcelona (Spain), and it is composed of 17 men who reported identifying with the concept of NAM [8] and who regularly meet once a month to discuss topics connected with masculinity. Thus, as we introduced in the previous section, they are used to debating scientific articles aimed at identifying strategies to combat chauvinism in different societies [50]. In these meetings, they also discuss the different interventions that are being carried out, such as workshops with adolescents and conferences that address citizenship. In addition to these men, there are 7 other men who sporadically collaborate in different activities organized by the association. Therefore, from a sample of 24 men involved in Men in Dialogue, we obtained 15 responses (62.5%). Table 1 includes ages on these 15 men (Mean age = 37.53, SD = 4.78; Mdn = 40.00); 86.6% of them had high levels of education (doctorate, graduate degree), and 13.4% had only finished secondary education. Some participants had careers in skilled professions, such as researchers and teachers, while others performed jobs requiring fewer skills like turner or production worker.

To meet ethical requirements for the data collection process, informed consent was obtained from all the participants before being interviewed. Additionally, confidentiality was guaranteed because interviewees’ names were anonymized and replaced by a pseudonym. Hence, statements given by members of Men in Dialogue could not be identified. Table 1 contains the pseudonym and age of each participant. The study was also validated by the Ethical Board of the Community of Researchers on Excellence for All on 25 June 2020, receiving the identification code 20210102.

One of the main differences of communicative methodology and other methodological approaches is the relevance given to presenting the transformative purpose of the research to the participants [45]. Thus, to fulfill this premise in our investigation, an egalitarian dialogue was established from the very beginning with the members of the Men in Dialogue men’s group, whereby the purpose of the study to identify ways to overcome unhealthy practices was clearly exposed. Thus, an initial meeting was arranged with some members of the association to inform them about this purpose. Then, to guarantee the profundity of their reflections, which is another relevant premise in communicative-oriented investigation, as well as to obtain answers from the highest possible number of people, we prepared a five-item open-ended questionnaire in Catalan (on Google Forms) that was sent to the members and collaborators of Men in Dialogue [51]. Overall, the timeframe for the data collection process was two months from the first meeting with the association to the implementation of the open-ended questionnaire. Then, we spent one month analyzing the data and contrasting the findings with the members of the men’s group who participated in the research.

The open-ended questionnaire included questions and sub-questions that sought to address our initial hypothesis: Friendships developed in movements linked to new alternative masculinities improve men’s mental and physical health. In this regard, the aforementioned questions are intended to provide qualitative data on the influence of friendship, as perceived by Men in Dialogue, on men’s happiness and physical health. We present here five open questions as they appeared in our questionnaire. They refer to key issues concerning men’s health and friendship:

1. Do you take care of your health? If yes, how do you handle this issue? Through what mechanisms?
2. What types of healthy habits do you follow during the week (e.g., diets, sports practice, checking information about food supplements, meals)? Why do you follow them, and with what purpose?
3. Do you talk about health issues with your male friends in Men in Dialogue or other spaces? What type of conversations do you have? Can you give an example? Is there solidarity in this area? And competitiveness? Why?
4. Has friendship with a guy or the guys of Men in Dialogue or other spaces helped you to improve your personal life (e.g., affective and sexual relationships, happiness)? How did they help you? Most importantly, has this had a later impact on your health habits (physical and mental)? If possible, can you explain this change (through what types of interactions)?
5. Can you explain any bad experience (friendship or toxic relationship) with a man that led to an opposite situation?

### 2.2. Data Analysis

The analysis of the data collected from the questionnaire was based on the long explanations that the participants provided online. Drawing on this information, firstly, a deductive coding process was begun that considered the communicative methodological approach described above and aimed at ensuring the anonymization of the information collected. To this end, the research team involved in the data analysis met regularly to select the quotes that responded to two kinds of dimensions that are the foundations of the communicative methodology: exclusionary and transformative [46,52]. The first kind are the barriers faced by people in the course of their lives that make it difficult to benefit from any correction or social provision. Conversely, the latter are the dimensions that contribute to overcoming such barriers. Based on this dichotomy, different issues linked to masculine friendship and health habits were inductively identified that had an exclusionary or transformative nature. Thus, we started by reading all the responses and inductively developing a categorization. After that, we selected and divided the quotes into two main categories: (a) Friendships that influence men to follow practices with adverse outcomes (Exclusionary dimensions) and (b) Friendships framed in the Men in Dialogue men’s group that encourage men to follow favorable practices (Transformative Dimensions). Five more subcategories were established considering this antagonistic dichotomy, which were: (1) self-undervaluation and lack of self-reflection; (2) competitiveness, relations of interest and superficiality; (3) sharing knowledge and solidarity; (4) conversations and interactions addressing favorable practices; and (5) evidence of the improvement of health status.

The research team involved in this article, with expertise in communicative-oriented analysis, established this codification. After that, the data codification and its interpretation were discussed with the members of Men in Dialogue who had participated in the research. This process added the participants’ reception of the findings to the results found by the research team.

## 3. Results

As mentioned above, transformative and exclusionary dimensions were considered to categorize the findings obtained in the qualitative open-ended questionnaire. In the present section, this distinction will be considered. First, the shaping of non-favorable masculine friendship and its influence on men’s health will be described, and second, the effects of favorable men’s friendship on mental and physical health will be detailed.

### 3.1. Friendship-Driving Practices with Adverse Outcomes

#### 3.1.1. Undervaluation and Lack of Self-Reflection

One of the behaviors that conditioned interviewed men to follow practices with adverse health outcomes is related to the self-undervaluation. This is how Robert explained his experience in this regard: “In general, toxic or meaningless relationships have led me to have unhealthy habits because a positive image of myself was lost”.

The men’s responses confirm that men do not disconnect this type of friendship from emotional well-being. In fact, they affirmed that in such relationships, emotions are not prioritized and are replaced by practices such as partying and consuming drugs. Oriach attested to this when he reflected upon what he was feeling and doing during a period of his life when he had friendships that influenced him to unfavorable health practices: “I did not take care of my emotions in periods of sadness, and I tried to forget them by replacing them with partying; I made little analysis about what occurs in oneself”.

#### 3.1.2. Competitiveness, Relations of Interest and Superficiality

Competitiveness was another of the characteristics emphasized by the participants in the study when they explained their previous male friendships. For instance, Esteve stated that interactions framed on competitiveness caused him a permanent state of anxiety, which affected his mental and physical health: “In general, my previous relationships were based on competitiveness. This caused me constant dissatisfaction that generated permanent anxiety. This state directly affected my mental and physical health”.

Similar effects are highlighted in Mariano’s story which is characterized by relations of interest that derived into bullying practices promoted by his friendships. This situation shaped his self-image and health habits because he was overweight, and his friendships mocked him for it. Considering Mariano’s words, this bullying affected his behavior and he acted violently. In addition, these interactions influenced him to not maintain an active lifestyle.

“In this sense, they made me feel bad about myself on the one hand, and, on the other hand, I responded with violence more than once, understanding that it was the only possible way to make me worthwhile. None of these relationships made me take care of myself. In addition, I think that it is a sensation that still affects me today, a misunderstood rebellion against that which assists you and the rejection of those friends who ‘take care of you”.

Lastly, Pedro’s story illustrates how superficiality is another element within friendship interactions which worsened his health habits and practices. He explained how nightlife was prioritized in front of other kinds of practices, such as sharing information that could have helped him in that moment:

“My previous friendships with guys have been quite linked to nightlife—partying—and maintaining unhealthy habits. In addition, we did not share information about how to be better or how to take care of ourselves. The focus was mainly going out to party”.

### 3.2. Friendships That Encourages Healthy Habits

One of the common issues that the participants stressed in the study was the importance of initiating masculine friendships that encourage healthy practices. All 15 of the respondents said that this type of friendship has improved their lives, increasing their happiness, and positively influenced their health habits. In the framework of their involvement or interest in Men in Dialogue, the quality of their friendships has changed intensely.

#### 3.2.1. Sharing Knowledge and Solidarity

By having different friendships from the past, most of the interviewees ensure that they are carrying out practices that are beneficial to their health. One of these practices is the sharing of useful knowledge. In the next quotation, Adrià emphasizes how he used to share information connected with food habits, myths about food and plastic toxicity.

“Yes, we have conversations about health. Basically, we talk about how to organize ourselves to have time to do sports and about some food and eating habits and we share other types of information, such as false myths about issues such as food. Another example is one day when we met, some of us had searched for information about the toxicity of plastics in our daily life and shared it with the rest of the group. In general, there is solidarity and not competition. I think it is because of the awareness that sharing this information makes everyone better, even including oneself. There is also the factor that previous conversations have created connections that make us act through solidarity”.

Solidarity is another pillar for the improvement as it was mentioned by several respondents like Adrià. He usually shares with some of his friends of Men in Dialogue the development of practices that have as an objective the improvement of men’s health habits. By doing so, Adrià affirms the existence of an implicit and respectful control that is established among the men that helps them to maintain an active and healthy lifestyle.

“Yes, it often comes out in conversations, if it is complicated with the group because everyone has different priorities on this topic, and it is difficult to create dynamics in spaces with many people. I know that at the friend level, with some of them, we share this issue about taking care of us, we talk about it and it helps me a lot; the fact that we both are attentive, or take a moment to enjoy certain activities such as when we are playing fewer sports. The theme of solidarity with friends is shared”.

#### 3.2.2. Conversations and Interactions Addressing Favorable Practices

Responses to open-ended questionnaires provide significant data about the relevance of establishing conversations based on valid arguments to transform personal relationships. For instance, Esteve affirms that conversations he has had with his current friends have had an impact on his mental and physical health.

“Yes, it helped me to gain security by approaching daily issues in a positive way through conversations with friends. This has led to a general improvement in my health at all levels, starting with the mental and affecting the physical”.

Similarly, Bogdan affirms that, through these conversations with friends, he has learned to understand what is happening to him, that is, to better interpret his physical and mental status. He believes that these interactions have recovered meaning in his life, and now he is more satisfied with his personal decisions.

“Yes, clearly the friendships with a few guys helped me to improve my life at all levels. Before, I understood nothing of what was happening to me, and now I have gained a lot in this regard. This has had great mental repercussions; it has relaxed me a lot and now I approach things differently than before. In addition, I am much more pleased with my life and with my work, and this is mainly due to my friends. I think that, for this change, the most important aspects were the talks in different contexts, which always made me think and reflect on the important issues of life. The meaningful interactions are those that have helped me more in this process, which is never-ending and requires constant work”.

Along the same lines, Antonio highlights the role of Men in Dialogue in all these changes in his life, particularly how the conceptualization of new alternative masculinities has made this change possible. Antonio confirms that his participation in Men in Dialogue has conditioned his daily practices which are more connected with healthy dynamics:

“A group of men decided to deepen our masculinity, following the model of Men in Dialogue. I made two friends, and we chose to work on our relationship with the NAM model (new alternative masculinities) that we were learning. Each conversation improves our relationship, although there is always a path, and we do things that we never considered before, at least myself, until these relationships began. We continue studying, we are getting our master’s degrees together, we will dream in doctoral theses, we think about doing sports tests together, we find more accurate answers in the work and we position ourselves with more success, etc.”

The nature of these friendships is an aspect highlighted by Pedro when he explains the influence of having conversations about love and desire with friends. He maintains that these conversations promote the eradication of unhealthy habits and the strengthening of healthy practices at physical and mental level.

“Yes. Friendship with a specific friend has helped me improve my personal, affective and sexual relationships. I am happier. It has helped me mainly through conversations and talking very clearly of my wishes because I have them and the power to change them. This has had a key impact on my health, especially in the elimination of drug use, the increase in my physical activity and the increase in my concentration and mental health”.

#### 3.2.3. Evidence of the Improvement of Health Status

Jordi’s words evidence the emergence of several improvements in his health status. Jordi details how, since engaging in conversations with friends that are based on emotional affection, his health problems have disappeared. He insists that friendship has given him much self-confidence.

“The increase in self-confidence that these relationships have given me has generated improvements in the choice of friends, knowing how to situate relationships, and improvements in desire, increased passion in relationships with friends and as a couple... Previously, I had some problems with my stomach and allergies that I have seen drastically reduced. The improvements I have made have been associated with my friendships that have given me security”.

The issue of self-confidence was also emphasized by three of the interviewees as being strongly linked to improvement in their health. For instance, Mariano notes that the confidence given by friends has influenced his change in regard to ending negative habits, such as smoking and being overweight.

“Yes, totally. My friendships are key to the improvement of my health: stopping smoking, taking more care of everything, and feeling capable of overcoming my health problems, especially being overweight. Everything about trust, understanding and living that the other person tells you is because they really care about you, and you feel that you want to be better, more handsome, happier, freer, etc. You have true friends who value you and want you to be better every day, and they help you to be better”.

It is noteworthy that the transformative elements presented in these sections reveal the possibility of opening new understandings about the effects of friendship, framed in the daily interactions of an egalitarian men’s movement, in men’s health habits and practices.

## 4. Conclusions

The review of the previous literature on the role of men’s friendship in health issues pays scarce attention on the effects of participation in an egalitarian men’s movement [2,22,41]. In fact, the existing investigation in this line of research is mostly focused on identifying the influence of friendship networks on men’s emotional well-being [22,53]. Thus, with the present article, we shed light on this topic, providing new insights into the relevance of fostering masculine friendships based on principles which drive men to follow healthy habits.

First, we can observe that our findings confirm previous studies on men’s friendship by showing how negative behaviors are encouraged when inconvenient friendships are fostered [54,55]. These kinds of relationships are framed by gender roles that strengthen the model of hegemonic masculinity which is commonly linked to risky behaviors [25,37]. These kinds of behaviors are mostly linked with the consumption of alcohol or drugs, but there are also studies which stress the negative impact on mental and physical health of perpetrating violence [3,11].

Second, considering our hypothesis that friendships developed in movements linked to new alternative masculinities are improving men’s mental and physical health, in this study preliminary support is provided with the data collected through the case study of the Men in Dialogue men’s group. In this regard, several common elements emerge that define men’s friendship in that group: (a) solidarity, (b) conversations and interactions addressing favorable practices, and (c) health improvement. In relation to solidarity, the men who participated in the investigation expressed the predominance of sincere relationships based on mutual help and sharing information. This dynamic has been confirmed as being very positive for the prevention of unhealthy habits and for the development of better physical and mental health. For instance, participants in the research confirmed having fewer problems with their weight, some of them have decided to not smoke, and there are other interviewees who are happier now than before being involved in Men in Dialogue. Concerning the shaping of conversations and interactions addressing favorable practices [56,57], the fieldwork illustrates the importance of establishing relationships, framed on support and trust that foster healthy practices and reinforce emotional well-being. Thus, the results show the interconnection between this emotional well-being and men’s physical health. Finally, findings also demonstrate significant progress into men’s lifestyle thanks to their participation in Men in Dialogue. This participation helped them to be more self-confident and to reject unhealthy habits. As a result, these men have started to perform sport routines, to follow healthy diets and to have positive beliefs about their personal life.

Although these conclusions are stimulating for the future of men’s studies and men’s lives, more research in this direction should be developed. The present study has some limitations; for instance, results are limited by the fact that they are mostly based on a quite homogenous sample of well-educated, middle-aged men living in Spain. Therefore, these results may not generalize to other groups of men. Similarly, there is another limitation that concerns the subjectivity of the participants’ reflections, which are focused on their feelings about the impact of Men in Dialogue on their health status. Thus, drawing on this interpretive nature, the results achieved in our study cannot be transferred to other egalitarian men’s movements.

## Figures and Tables

**Table 1:** Interviewees’ age.

Pseudonym	Age	Pseudonym	Age
Bogdan	32	Pedro	32
Adrià	34	Mariano	42
Jordi	40	Sebastià	32
Carlos	41	Robert	41
Pere	29	Albert	41
Antonio	43	Oriach	34
Esteve	43	Sandro	40
Luis	39		

## Data Availability

The data presented in this study are available on request from the corresponding author. The data are not publicly available due to ethical restrictions.
